# Transnasal humidified rapid-insufflation ventilatory exchange (‘THRIVE’) in the coronavirus disease 2019 pandemic

**DOI:** 10.1017/S0022215120002753

**Published:** 2021-01-07

**Authors:** S Y Hey, P Milligan, R M Adamson, I J Nixon, A F McNarry

**Affiliations:** 1 Department of Otolaryngology and Head and Neck Surgery, NHS Lothian, Edinburgh, UK; 2 Department of Anaesthesia, NHS Lothian, Edinburgh, UK

**Keywords:** Airway Management, Anesthesia, Continuous Positive Airway Pressure, COVID-19, Insufflation, Intubation, Larynx, Oxygen, Trachea

## Abstract

**Background:**

Since the start of the coronavirus disease 2019 pandemic, transnasal humidified rapid-insufflation ventilatory exchange (‘THRIVE’) has been classified as a high-risk aerosol-generating procedure and is strongly discouraged, despite a lack of conclusive evidence on its safety.

**Methods:**

This study aimed to investigate the safety of transnasal humidified rapid-insufflation ventilatory exchange usage and its impact on staff members. A prospective study was conducted on all transnasal humidified rapid-insufflation ventilatory exchange cases performed in our unit between March and July 2020.

**Results:**

During the study period, 18 patients with a variety of airway pathologies were successfully managed with transnasal humidified rapid-insufflation ventilatory exchange. For each case, 7–10 staff members were present. Appropriate personal protective equipment protocols were strictly implemented and adhered to. None of the staff involved reported symptoms or tested positive for coronavirus disease 2019, up to at least a month following their exposure to transnasal humidified rapid-insufflation ventilatory exchange.

**Conclusion:**

With strictly correct personal protective equipment use, transnasal humidified rapid-insufflation ventilatory exchange can be safely employed for carefully selected patients in the current pandemic, without jeopardising the health and safety of the ENT and anaesthetic workforce.

## Introduction

The ENT UK guidelines for changes in ENT during the coronavirus disease 2019 (Covid-19) pandemic, published in March 2020, are centred on the protection of our ENT workforce from nosocomial transmission of the severe acute respiratory syndrome coronavirus-2 (SARS-CoV-2).^1^ As all airway surgery may be aerosol-generating and any patient may harbour infection, all staff involved in such cases are at potential risk.^2^ Various publications have further addressed the safety concerns and implications of the pandemic on airway service provision from both ENT and anaesthetic perspectives.^3–5^

Transnasal humidified rapid-insufflation ventilatory exchange (‘THRIVE’) has been classified as a high-risk aerosol-generating procedure and is strongly discouraged by several sources.^6–8^ In addition, a worldwide shortage of medical oxygen supply had further prompted scrutiny around the use of high-flow nasal oxygen. While high-flow nasal oxygen has been used in many cohorted intensive care units, the lack of conclusive evidence on the safety of transnasal humidified rapid-insufflation ventilatory exchange continues to be a matter of debate among airway practitioners.^2^

## Case report

In our unit, we have continued to utilise transnasal humidified rapid-insufflation ventilatory exchange in selected cases, following careful airway risk assessment and shared decision-making.

**Fig. 1.**
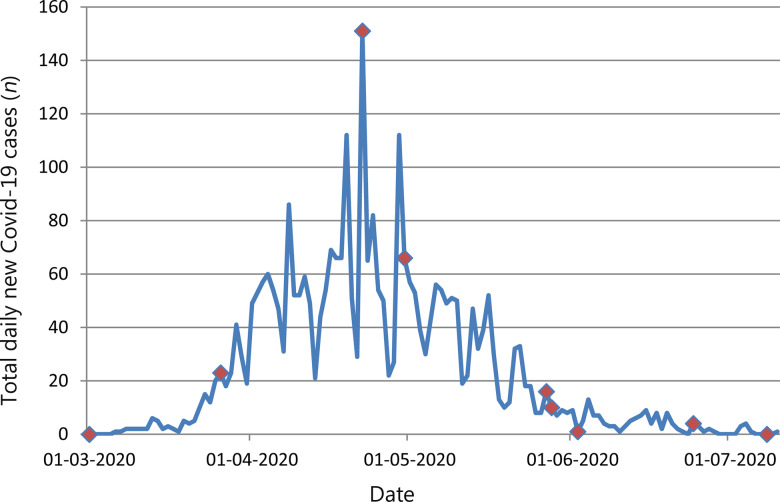
Total daily new cases of coronavirus disease 2019 (Covid-19) in NHS Lothian and dates (red-dotted) on which transnasal humidified rapid-insufflation ventilatory exchange (‘THRIVE’) was performed in our unit. (Data courtesy of John Frace, University of Highlands and Islands, Scotland.)

In keeping with current guidelines, we report the successful management of 18 patients who underwent transnasal humidified rapid-insufflation ventilatory exchange from March to July 2020 (Figure 1).

For each case, there were 7–10 staff members present, including operating theatre nurses, anaesthetists and surgeons. Appropriate personal protective equipment (PPE) protocols, such as use of a fitted filtering facepiece code 3 (FFP3) mask, fluid-resistant gown, gloves and eye shields, were strictly implemented and adhered to by all staff members in attendance. None of the staff involved reported symptoms or tested positive for Covid-19 following their exposure to transnasal humidified rapid-insufflation ventilatory exchange. This was observed for up to at least a month following their participation in any transnasal humidified rapid-insufflation ventilatory exchange cases.

## Discussion

The most recent guidance on airway management for the endemic phase of Covid-19 suggests that use of high-flow nasal oxygen should be considered relatively, rather than absolutely, contraindicated.^9^ We have previously reported its benefit in patients for whom attempted conventional intubation may be traumatic or dangerous.^10^ Patients with conditions such as subglottic stenosis have not disappeared during the pandemic, and we continue to provide ENT emergency and oncology services. Challenging airways are not uncommonly encountered, highlighting the potentially beneficial role of transnasal humidified rapid-insufflation ventilatory exchange.

While our cohort is limited in size, the non-availability of regular and routine testing of asymptomatic staff at our local trust precludes comments on the possibility of any subclinical infection, or the infection of patients by staff members. Nonetheless, with the ongoing lack of conclusive evidence, our observational outcome suggests that with strictly correct PPE use, transnasal humidified rapid-insufflation ventilatory exchange can, in carefully selected patients, be safely used during the current pandemic, without jeopardising the health and safety of the ENT and anaesthetic workforce.
